# Sesame, Pistachio, and Macadamia Nut: Development and Validation of New Allergenic Systems for Fast Real-Time PCR Application

**DOI:** 10.3390/foods9081085

**Published:** 2020-08-08

**Authors:** Martina Torricelli, Elisa Pierboni, Cristina Rondini, Serena Altissimi, Naceur Haouet

**Affiliations:** Experimental Zooprophylactic Institute of Umbria and Marche Regions “Togo Rosati” (IZSUM), Via Salvemini 1, 06126 Perugia, Italy; schwalben56@gmail.com (C.R.); altiseri2020@gmail.com (S.A.); mn.haouet@izsum.it (N.H.)

**Keywords:** food allergen, fast real-time PCR, validation, commercial food products, allergy

## Abstract

Food allergy is a worldwide health problem that concerns infants to adults. The main health risk for sensitised individuals is due to the presence of traces of allergens as the result of an accidental contamination during food processing. The labelling of allergens such as sesame, pistachio, and macadamia nut on food products is mandatory according to Regulation (EU) N. 1169/2011; therefore, the development of suitable and specific analytical methodologies is advisable. The aim of this study was to perform a multi-allergen real-time PCR system that works well in fast mode at the same annealing temperature and with the same thermal profile. The real-time PCR was developed designing new, specific, and efficient primer and probe systems for the *2S albumin* *gene* for sesame and pistachio and for the *vicilin precursor* *gene* for macadamia nut. These systems were subjected to a robust intra-laboratory qualitative validation process prior to their application, by DNA extraction and fast real-time PCR, on some real market samples to reproduce a potential allergen contamination along the food chain. The developed system results were specific and robust, with a sensible limit of detection (0.005% for sesame; 0.004% for pistachio; 0.006% for macadamia nut). The performance and the reliability of the target systems were confirmed on commercial food samples. This molecular approach could be used as a screening or as a support tool, in association with the other widespread monitoring techniques (such as ELISA).

## 1. Introduction

Food allergies are considered a relevant public health problem that affects over 220 million people worldwide [1,2]. They are further considered, after respiratory allergies, a ‘second wave’ of the allergy epidemic [3], whose symptoms can vary from gastro-enteric disorders, respiratory symptoms (such as rhinitis and asthma), skin reactions (such as urticaria and atopic dermatitis), to life-threatening anaphylactic shock and death in sensitised individuals [4]. The prevalence of food allergy depends on genetics factors, origin country, cultural habits, and dietary habits, and it could be affected by the period of exposure to a certain allergen over a lifetime [5]. Although the avoidance of food allergens would be the only effective solution to protect the health of allergic individuals [6], the unintentional presence of these substances, so-called hidden allergens or undeclared allergens, is possible due to cross-contamination phenomena along the food chain and processing. On the other hand, in some cases, fraudulent substitutions can be also registered [7]. Furthermore, EU legislation requires mandatory labelling for allergenic food ingredients and Regulation (EU) N. 1169/2011 [8] provides a list of 14 substances or groups of substances commonly responsible for allergies or intolerances that have to be labelled and highlighted in the list of ingredients, independently from allergenic proteins presence and regardless of quantity.

On this basis, the development of suitable and specific analytical methodologies to ensure consumers protection and compliance with food labelling regulation is strictly necessary. The available approaches for the detection and quantification of food allergen are immunological, proteomics, and DNA-based tests, especially enzyme-linked immunosorbent assay (ELISA) and lateral flow device, mass spectrometry (MS) and polymerase chain reaction (PCR), respectively [9,10,11]. ELISA is the most widely recognised and applied technique in routine analytical control for its high sensitivity, low cost, ease of use, and rapid result acquisition. On the other hand, it shows some disadvantages based on the complexity of method development, the antibody quality (which depends also on batch), and cross-reactivity due to homology between the antibody recognition sites of different proteins, determining false positive results. Other limitations consist of the high frequency of false negatives relative to the matrix effect and protein denaturation or degradation in food processing with high temperature and pressure treatment and no possibility of analysis in a multiplex format [12,13]. Over the last few years, MS offers an approach of resonant interest as a potential confirmatory and direct quantitative method that can detect simultaneously multiple allergens [14,15]. However, it requires expensive equipment and highly trained analysts before a quantitative approach can be achieved. DNA-based detection methods, such as real-time PCR, are alternative methods for allergen detection that have attracted the attention of researchers in this field. In this way, real-time PCR technology has proved to be very effective for the detection of allergenic foods [16]. DNA molecules are more stable than protein and are more resistant to chemical and physical treatments to which processed food are subjected [17]: for this reason, DNA-based tests are less affected by matrix effects than ELISA. The PCR, especially real-time PCR such as the TaqMan assay, is a very specific and sensitive tool. The specificity and sensitivity are due to the use of specific primers and probes that, in allergen analysis, recognise and amplify the allergen coding region or other constitutive *genes* of the target as an indirect method [18].

The aim of the study was to implement the panel of food allergens already investigated in our control laboratory (soybean and peanut) [18]. Therefore, the main objective was to identify systems that worked well with the same annealing temperature to carry out a multi-analysis, adopting a TaqMan chemistry with the same thermal profile, a unique reaction mix, and an optimised preparatory phase in order to detect low and specific quantities of allergens with a detailed and extensive investigation among related species.

From an accurate literature investigation, the real-time PCR systems for sesame, pistachio, and macadamia nut did not satisfy the established parameters.

Sesame *(Sesamum indicum L.)* belongs to the Pedaliaceae family. Roasted sesame seed could be found in different types of food such as bakery products (snacks, breads, biscuits), processed meat, fast foods, vegetarian food, and ethnic dishes, or it could be consumed in the form of oil used for cooking above all in Oriental, Chinese, and South American cuisines [19,20,21]. Sesame has always been considered one of the major causes of food allergy [22,23,24], and moreover, it is increasing in some countries such as Israel and France, where the highest prevalence was observed [21]. Furthermore, it is possible to observe a cross-reaction between sesame seeds and tree nut or peanut, and thus in the same way cause an allergic reaction in affected individuals [25].

To date, some of the allergenic proteins of sesame have been identified [19,26], some of them common to other allergens, in particular a sulfur-poor 2S albumin (Ses i 1) and a sulfur-rich 2S albumin (Ses i 2), but also a 7S vicilin-like globulin (Ses i 3), [27,28], two oleosins (Ses i 4; Ses i 5) [29], and two 11S globulins (Ses i 6 and Ses i 7) [30]. Until now, the majority of the studies on genomic DNA were conducted on *2S albumin gene* (NCBI acc. No. AF240005), obtaining variable limit of detection (LOD) values [26,31,32,33]. Koppel et al., 2010 and 2012 [34,35] conducted 2 hexaplex real-time PCR studies and investigated also the *oleosin gene* (NCBI acc. No. U97700), while Zhang et al., 2018 [36] studied *2S albumin* in SYBR green assay. Furthermore, Lopez-Calleja et al., 2015 [20] applied PCR systems on an internal transcribed spacer (ITS) region (NCBI acc. No. AF169853), while Ehlert et al., 2009 [37] used a ligation-dependent probe amplification (LPA) multiplex system for the simultaneous detection of DNA from 10 different food allergens. In addition, Lopez-Calleja et al., 2017 [38] describes a multiplex ligation-dependent probe amplification (MLPA) technique for the simultaneous detection of five food allergens: sunflower, poppy, flaxseed, sesame, and soy.

Pistachio belongs to the Anacardiaceae family together with cashew and mango, and allergenic cross-reactivity between these species could be registered. Pistachio nut, the fruits of the pistachio tree *(Pistacia vera)*, is an edible tree nut; the kernels can also found in ice cream, confectionery, and other gourmet products or can often be eaten whole, either fresh or roasted and salted. The fraudulent substitution of pistachio nuts is not very frequent because of the high price and its presence also in the form of a lower-quality variety in processed products is usually due to adventitious contamination [39]. To date, five proteins have been characterised and identified as allergens in the World Health Organisation/International Union of Immunological Societies WHO-IUIS list (WHO/IUIS Allergen Nomenclature Database): Pis v1, Pis v2, Pis v3, Pis v4, and Pis v5. Pis v4 is a manganese superoxide dismutase [40], and the rest of them represent the major seed storage protein constituents of the nuts [41]. Pis v1, Pis v2, and Pis v4 are considered and proposed as major allergens, while Pis v3 and Pis v5 are considered and proposed as minor. The potential cross-reactivity of pistachio with cashew nut is well known and demonstrated also by the molecular homology. In particular, Pis v1 (NCBI acc. No. ABG73108) presents a 66% amino acid sequence identity in 97% of the query cover with Ana o 3 (cashew) (NCBI acc. No. AAL916655) [42]. Furthermore, Pis v3 is characterised by the high homology with Ana o 1 from cashew nut (80% identity, 90% similarity), highlighting the “likelihood” of considerable cross-reactivity between these two allergens [42]. To date, some DNA-based methods are available in the literature for the detection and/or quantification of pistachio allergens. In particular there are three methods: a qualitative PCR end point [43] with a limit of detection of 100 mg/kg, an LPA multiplex system [37], and a multiplex PCR based on hydrolysis probe based on a *dehydrin* (*Cor gene*) (NCBI acc. No. Y07600) [35], with a relative limit of detection (LOD) of about 0.1%, but with a not specific signal for cashew nut. Internal transcribed spacer (ITS) between 18S ribosomal RNA and 5.8S ribosomal *genes* (NCBI acc. No. AY677201) was the target *gene* of two real-time PCR with the hydrolysis/TaqMan probe method [38,44], obtaining a sensitivity level and a relative LOD of 4 mg/kg of pistachio in cookies and 0.1 mg/kg of pistachio nut DNA, respectively. Finally, Pis v 1 (NCBI acc. No. DQ631675.1) was the target *gene* in three methods: a multiplex PCR coupled to capillary electrophoresis [45] with a relative LOD of 0.005% of pistachio in maize powder, and a SYBR-Green and locked nucleic acid (LNA) probe-based real-time PCR with an LOD of 100 mg/kg and 10 mg/kg, respectively [38]. For the considered systems, no cross-reactivity with the analysed plant or animal species was registered.

At last, macadamia nuts, belonging to Proteaceae family, are shelled kernels of the fruits of two species, namely *Macadamia integrifolia* (Maiden and Betche) and *Macadamia tetraphylla* (L.A.S Johnson), of commercial importance. Macadamia nuts are appreciated for their organoleptic properties and are largely consumed either directly, roasted, or as a component of confectionery products such as filled chocolates or nougats [46]. Fraudulent substitution is an improbable event due to the considerable high price of macadamia nut: despite this, adventitious cross-contaminations may occur when various nuts, also of lower quality, are used in the same food processing facility, sharing the same equipment. However, currently, few publications are available in the literature for macadamia detection, in particular: Brežná et al., 2009 [47] and Ehlert et al., 2009 [37] used the *vicilin precursor gene* (NCBI acc. No. AF161883) as the target for TaqMan real-time PCR and LPA systems, respectively; López-Calleja et al., 2015 [20] and Ito et al., 2018 [48] applied the ITS multi-copy *gene* in real-time PCR and TB green (intercalator-based reagent) singleplex/tetraplex PCR, respectively. All the methods exhibited a 100% of specificity, even if Brežná et al., 2009 [47] tested an exclusivity panel of 16 plant species and without including the pecan nut. LOD values obtained in the different studies ranged from 1.45 pg (practical LOD: 0.02%) [47] to 0.1 mg/kg [20] and to 1 pg [48].

From the bibliographical investigation, we decided to adopt systems based on allergenic targets, in particular, the *2S albumin gene* for sesame and pistachio and the *vicilin precursor gene* for macadamia nut, rather than internal transcribed spacer (ITS), which is characterised by species variability. To meet the project aims, it was necessary to develop and validate new systems for pistachio, sesame, and macadamia nut allergens, which were extracted with an efficient and “food field-universal” extraction protocol [18]. Another objective was to reduce the amplification time, adopting the real-time PCR in a fast mode process. Following the optimisation of specific and sensitive analysis, these developed methods were applied for the detection of allergenic ingredients in some commercial food samples, in order to reproduce and to evaluate possible and accidental contamination along the food chain, always with the final purpose of safeguarding consumer health.

## 2. Materials and Methods

### 2.1. Sample Selection and Reference Materials

Commercial sesame seeds, pistachio, macadamia nut, and the other samples used in the experimental tests were purchased in a local supermarket. Some samples were milled with Grindomix GM200 (Retsch, Haan, Germany), adding dry ice to avoid melting and aggregation in high-fat products. All the ground samples were conveniently homogenised using the plastic bag technique [49]. Nuts, seeds, leaves, and fruits used also for the specificity test were collected and ground under liquid nitrogen with a mortar and pestle. Animal DNAs used in the specificity test were supplied from a Veterinary Diagnostic Laboratory of IZSUM Institute. SureFood^®^ QUANTARD Allergen 40 (R-Biopharm, CONGEN Biotechnologie GmbH, Berlin, Germany) and Allergen RM 800 (BIOTECON Diagnostics, GmbH Hermannswerder, Germany) were used as positive controls for both the DNA extraction and the real-time PCR.

### 2.2. DNA Extraction and Assessment

DNA was extracted twice according to the protocol published in Pierboni et al., 2018 [18], which was based on CTAB (hexadecyltrimethylammonium bromide) extraction buffer [50], combined with NucleoSpin gDNA Clean-up (Macherey Nagel^®^, Duren, Germany) for DNA purification. The starting material for DNA extraction was 2 ± 0.2 g for not homogenised matrices and 0.2 ± 0.02 g for uniform ones. To obtain a higher yield, DNA was concentrated and eluted in 50 µL of elution buffer (DE), as also reported in Pierboni et al., 2018 [18]. The extracted DNA was stored at +4 °C for a week or −20 °C for a longer time. Once extracted, the DNA concentration of the two replicates for each sample was established fluorimetrically by the mean of a Qubit dsDNA BR Assay Kit (Thermo Fisher Scientific, Waltham, MA, USA) in the Eppendorf BioSpectrometer (Eppendorf, Hamburg, Germany). DNA quantity was also represented by the Cq (quantification cycle) value; the limit of acceptability of data repeatability is based on a Cq difference of a maximum of 0.5 between replicates [51]. DNA quality was assessed using an inhibition test [52,53] in fast real-time PCR, targeting the actin *gene* [54]. The test is based on the analysis of two replicates of undiluted DNA, and its dilution was 1:4 for each extracted sample. If inhibitors are absent, the difference between the measured mean cycle threshold (Cq) of undiluted and diluted DNA (ΔCq) should be of 2, with an acceptability range of 1.5 to 2.5.

### 2.3. Oligonucleotides

The primers and probes used in this study were synthesised by Invitrogen (Thermo Fisher Scientific, Waltham, MA, USA) and Metabion (Matabion International AG, Planegg, Germany) for BHQ1 quencher; details are reported in Table 1. For the three investigated allergens, the set of new primer and probe oligonucleotides were selected and designed using Primer Express software v. 3.0 (Applied Biosystems^®^, Foster City, CA USA), which is a specific tool to provide the best qPCR assay design on the basis of different considered parameters and of the lower penalty. For pistachio, two sets of oligonucleotides, designed on two different regions of the target *gene* and with differently labelled probes, were assessed. The amplicon length for sesame, pistachio (a), pistachio (b), and macadamia nut were 77 bp, 62 bp, 59 bp, and 78 bp, respectively.

### 2.4. Real-Time PCR

Real-time PCR was run in fast mode by a 7900HT Fast Real-Time PCR System (Thermo Fisher Scientific, Waltham, MA, USA), using the following cycling conditions and thermal profile: 95 °C for 20 s, 40 cycles with 95 °C for 3 s, and 60 °C for 30 s. The reaction volume was 20 µL, with 1X of TaqMan^®^ Fast Universal PCR Master Mix (2X) No AmpErase UNG (Thermo Fisher Scientific, Waltham, MA, USA), 2 µL of DNA, and primers/probes at concentrations obtained in optimisation test. Data analysis was performed by SDS 2.4 software (Applied Biosystems^®^, Foster City, CA USA). Each PCR run included not template control (NTC) and positive amplification control.

### 2.5. Optimisation of Primer and Probe Concentrations

For the optimisation of sesame primer and probe concentrations, about 200 hge (haploid genome equivalents), corresponding to about 0.2 ng of DNA, was tested assuming that the haploid genome size (1C) is 0.97 pg [55,56]. For pistachio and macadamia nut, information about haploid genome size is not available, so the test was carried out using about 2 ng and 0.2 ng of DNA, respectively. For each set of oligonucleotides, nine combinations of three concentrations of 150, 300, and 900 nM in four replicates were performed. For each probe, four concentrations of 100, 150, 200, and 250 nM in four replicates were tested and evaluated. The best primer concentrations corresponded to the combination with the higher ∆Rn, taking into account also the lowest Cq and standard deviation, while the best probe concentration was chosen considering as the first parameter the lowest Cq and the lowest standard deviation and successively the highest ∆Rn value, as recommended by the manual protocol of the TaqMan^®^ Universal PCR Master Mix.

### 2.6. Specificity

During the design, all primers and probes were successfully checked in silico for relevant homologies by the Blast program on the Nucleotide Collection (nr/nt) sequence database, (BLASTN) within the GenBank databases in the NCBI (National Center for Biotechnology Information). In vitro, the specificity was checked experimentally in a wide exclusivity panel of a total of 31 samples, including also allergen and botanical closely related species; these samples were tested in duplicate with concentrations adjusted on Cq 25–28 for the most of the samples and on Cq 29–32 for those DNA that were more difficultly extracted (especially nuts and pink pepper), whose Cq values were obtained by actin fast real-time PCR inhibition assay [54]. Each system was tested also for inclusivity, in particular on the extracted DNA of sesame, pistachio, and macadamia nut species.

### 2.7. LOD, Amplification Efficiency, and Linearity

The LOD of sesame, pistachio, and macadamia nut methods was evaluated testing 9 concentrations series of DNA in decreasing ng and/or hge, each in 10 replicates. The first point of dilution corresponded to 320 hge or 0.31 ng, 0.12 ng, and 0.8 ng, respectively for sesame, pistachio, and macadamia nut systems. The last dilution, where all 10 results gave amplification, was defined LOD_10_ and was verified in 60 replicates together with the upstream or downstream dilution to define LOD_95_ or the absolute LOD of the method, corresponding to a dilution point with at least 59/60 amplification signals. The amplification efficiency and linearity were represented respectively by slope and correlation coefficient R^2^ of the regression line obtained with the first five dilutions used in the LOD_10_ test [53,57,58].

### 2.8. Robustness

In robustness assessment, the target sequence copy numbers or ng corresponds to the LOD value of the method multiplied by three [59]. A multi-factorial experiment design was carried out in triplicate, setting up two PCR runs: run 1 for the reference method and run 2 (A and B) on a different thermal cycler, which is a 7500 Fast Real-Time PCR System (Applied Biosystems^®^, Foster City, CA USA), with different master mix concentrations (−10%), different primer and probe concentrations (−30%), different reaction volumes (A: +1 µL; B: −1 µL), and with an annealing temperature farther from the Tm (melting temperature) of primers [58].

### 2.9. Commercial Food Samples

In order to test and verify the feasibility of the validated systems for sesame, pistachio, and macadamia nut, some different products were collected at a local supermarket, selecting samples with PAL (precautionary allergen labelling); samples declaring the presence of allergen on the label; and samples not declaring the presence of allergen on the label. As no commercial products stating the presence of macadamia nut on the label were found in the local supermarket, spiked samples were prepared. The different products were ground with Grindomix GM200, homogenised, and then 0.2 g of macadamia nut were added to 1.8 g of starting sample. The matrix effect was also evaluated, co-extracting 0.2 g of macadamia nut as control. DNA extracted twice from the samples were assessed by real-time PCR for the allergen target *gene*, testing one of each extract, after the inhibition test.

## 3. Results and Discussion

### 3.1. Quantity and Quality Assessment of Extracted DNA

DNA extracted from sesame, pistachio, and macadamia nut and from the collected commercial food samples were evaluated by the fluorometer, showing a final concentration in the range between about 40 and 100 ng/μL. The DNA concentration of the other samples used for the specificity test was always measured with a fluorometer and varied from about 10 to 200 ng/μL, depending on the type of matrix, where the lower values were obtained for leaves, seeds, and dried fruits. The lower concentrations concerned only a few species belonging to the various categories (cashew nut, mango, linseed, and celery leaves), which was probably due to the different variety, variable genome size, and components. The inhibition test carried out by fast real-time PCR for the actin *gene* revealed that the mean difference (ΔCq) between the measured mean Cq of undiluted DNA and its dilution 1:4 was in accordance with the parameters [52,53]. As deduced from the data reported in Supplementary Table S1, for the targets used in the specificity test and for the allergens that are the object of this study, good results were obtained in terms of actin Cq and ΔCq for plant species, so in terms of DNA quantity and quality, indicating a valid and performing extraction method. As regards DNA extracted from commercial food samples, actin values of mean Cq between two extracts (Table 2) highlighted a certain variability depending on the type of matrix. In particular, the matrices that were more complex and difficult to extract, showing a higher actin Cq (34.02–35.54 Cq), were milk chocolate, pistachio ice cream, and 4 nuts cream. A minor repeatability between the two extracts was observed in the 4 nuts cream and snack bar. The quality of extracted DNA expressed as ΔCq was good for all the matrices, except for the 4 nuts cream and the mixed nuts, which were close to the acceptability limit of 1.5. Anyway, for all the analysed commercial foods, both qualitative and quantitative results were valid and in line with the acceptance criteria. In all real-time PCR runs, positive and negative extraction and amplification controls were tested, resulting as expected.

### 3.2. Assay Optimisation

Variation of the primers and probe concentrations was tested in order to provide optimal assay performance. The primers concentration of sesame target (*2S albumin*), pistachio (a, b) *gene* (*2S albumin*), and macadamia nut target (*vicilin-AMP*) was selected according to the best parameters reported in the Materials and Methods section, and these were 900/900 nM, 900/150 nM, and 900/900 nM respectively for primer forward and reverse. The probe concentrations of sesame, pistachio (a, b), and macadamia nut were respectively 150 nM, 200 nM, and 250 nM. For probe selection, among the comparable different concentrations, also the reagent cost factor was taken into account. Data and the results relative to mean Cq, a standard deviation of repeatability (SDr), and ΔRn are listed in Supplementary Table S2.

### 3.3. Assay Validation

No false negative or false positive occurred from the real-time PCR runs, so specificity was considered 100% either for sesame, pistachio, or macadamia nut systems (Table 3). Testing especially the allergen target systems against other substances, the absence of signals was also supported by in silico verifications. The specificity results were good as well as the cross-reactivity absence, considering moreover that some of the allergens belong to the same family and are then phylogenetically close or may present molecular homologies (Supplementary Table S1).

The obtained limits of detection of sesame, pistachio (a, b), and macadamia nut systems are reported in Table 4. As regards LOD_95_ or absolute LOD, for sesame, 57/60 amplifications were obtained at 2 hge/0.002 ng, while 60/60 amplification signals were registered at 5 hge/0.005 ng. For macadamia nut, 56/60 amplifications in a concentration of 0.003 ng, while there were 60/60 for 0.006 ng. The pistachio (a) system revealed 59/60 amplifications in a concentration of 0.004 ng but only 54/60 for 0.002 ng; in the same way, for the pistachio (b) system, 60/60 amplification signals were registered in a concentration of 0.004 ng, but there were only 52 for 0.002 ng.

Summarising, the practical LOD expressed in percentage was 0.005% for sesame, 0.004% for the two systems of pistachio, and 0.006% for macadamia nut. Considering these LOD values, it is possible to state that the developed real-time PCR systems are adequate, sensitive, and efficient also to detect very low quantities of the involved allergen.

The linearity of sesame, macadamia nut, and pistachio (a, b) was above 0.99. Concerning PCR efficiency, the best percentage was obtained for pistachio (a), specifically about 98%, and then around 90% for the macadamia system, about 86% for sesame, and 80% for pistachio (b). The differences in PCR efficiency may depend on the sequence position where the oligonucleotide set anneals, as demonstrated by pistachio system (a) compared to (b).

As shown in Table 5, for sesame, pistachio, and macadamia nut, the real-time systems designed on *2S-albumin* and *vicilin-AMP genes* appeared to be robust, with especially a concordance of 100% for the considered and analysed multi-factorial parameters [58,59].

Therefore, the behaviour of the two pistachio systems (a, b) was very similar, showing to be specific, sensitive, and robust at the same time. The only difference was observed in terms of efficiency, which was lower for the b system as also confirmed by a further experimental assessment.

### 3.4. Commercial Food Samples 

Table 2 shows the results of commercial food samples that were qualitatively in accordance with the labelled declarations. In the biscuits sample, late amplification signals were obtained for the pistachio (b) system, but not for the pistachio (a) system; therefore, they were considered not detected and thus in compliance with the label. For a product (“Tarallucci Salt snack”), unexpected amplification signals were obtained for pistachio with both systems (a, b) but with high and poorly repeatable Cq, over an estimated cut-off of 36.08 (LOD: 34.09 Cq + 2 SDr of 0.59) and 36.82 (LOD: 35.02 Cq + 2 SDr of 0.90), for systems (a) and (b), respectively. Therefore, this product was in compliance with label, but probably the matrix should be an object of further assessment regarding cross-contamination. In order to verify that the obtained signal does not derive from aspecificity and cross-reactivity with other plant species, the ingredients not evaluated in the specificity test (fennel and wheat flour) were tested in real-time PCR against pistachio systems, resulting in undetermined and then negative results.

From the analysis of samples spiked with macadamia nut, no relevant matrix effects were observed: indeed, the same extracted matrices showed similar mean actin Cq, except for the “Tarallucci Salt snack”, where 3 cycles of difference were obtained.

The spiked samples had lower repeatability than the relative matrices without macadamia nut, as highlighted by the higher standard deviation.

## 4. Conclusions

The aim of the work was to implement the panel of food allergens already investigated in our control laboratory (soybean and peanut) with new target systems that work at the same conditions. Therefore, the study presented new fast real-time PCR assays designed for sesame, pistachio, and macadamia nut. The developed system results were robust, specific, and sensitive, and the systems were able to work well in a fast mode, at the same annealing temperature and with the same thermal profile. These advantages allowed performing a multi-allergens assay and verifying different targets in the same analytic session, saving time and protecting consumer safety. With the adoption of a “food field-universal” in-house DNA extraction procedure, it was possible to extract DNA of good quality from a large variety of matrices, even complex ones, resulting in adequate performance. This methodology could be easily implemented in any analytical laboratory that performs routinely real-time PCR, and it could be used in food control, safeguarding both producers and consumers against the presence of hidden allergens. Last but not least, we focused this study on *genes* codifying allergen proteins present in single copy; however, they were not present in multi-copy in the cell, such as the ITS region, which could interfere with the uniformity of the results obtained for different varieties and/or cultivars of the same species. This approach can be useful both to overcome cross-contamination problems related to multi-copy targets and possibly to translate these methods in digital PCR. This new molecular approach allows providing a precise and accurate quantification of nucleic acids without the need of a standard curve, especially for low concentration and/or complex samples, reducing susceptibility to inhibitors [60]. Furthermore, digital PCR is suitable for multiplex analysis because the systems do not compete against the others [61]. In conclusion, the future goal is to translate the reliable systems in singleplex real-time PCR for a multiplex screening approach in digital PCR.

## Figures and Tables

**Table 1:** Target *genes* and sequence details.

Allergen	Target *gene*	GenBank	System Name	Oligonucleotide Sequence 5′-3′	Reference
sesame	*2S-albumin*	AF240005.1	ses-PG-F	AGTTCAGGTCCTGCCAGAGGTA	This work
			ses-PG-R	CATTTCCAGAACTTCATCCTCTTCA	
			ses-PG-P	FAM-TTGTCGCAAGGACGCAGCCCA-BHQ1	
pistachio_a	*2S-albumin*	DQ631675.1	pisa-PG-F	CCTATCTGCCTTCGCATTCC	This work
			pisa-PG-R	CCACAGTAGCGCGGTAGATG	
			pisa-PG-P	FAM-AGGCATTGGCCGCCAGGATG-BHQ1	
pistachio_b	*2S-albumin*	DQ631675.1	pisb-PG-F	CACTGCCAAATGTACGTGCAA	This work
			pisb-PG-R	GTGAGCGAGTGTCCGTCTTG	
			pisb-PG-P	FAM-CTCTTCTGGACCTCCT-MGB	
macadamia nut	*vicilin-AMP*	AF161883.1	mac-PG-F	GAGCCGTACCTCAGTACCTTCAG	This work
			mac-PG-R	CACCCCACGCAGCTTCTC	
			mac-PG-P	FAM- CGAGGCTGCGCTCAACACACAAAC-BHQ1	

AMP: *Macadamia integrifolia* antimicrobial peptide family protein (vicilin) precursor; BHQ: black hole quencher; FAM: 6-carboxyfluorescein; MGB: minor groove binder.

**Table 2:** Results of analysed commercial food samples.

Commercial Samples	System														
	Actin		Sesame				Pistachio						Macadamia Nut		
	Mean Cq	SDr	DiL	NDiL	PAL	Cq	DiL	NDiL	PAL	Cq (a)	Cq (b)	DiL	NDiL	PAL	Cq
vegeterian burger	22.84	0.44		x		n.d.		x		n.d.	n.d.		x		n.d.
integral toasted bread	25.25	0.11			x	n.d.		x		n.d.	n.d.		x		n.d.
cereal biscuits with yogurt	24.64	0.52		x		n.d.		x		n.d.	n.d.		x		n.d.
cous cous	23.24	0.91			x	n.d.		x					x		n.d.
mixed nuts	28.27	0.00		x					x					x	n.d.
muesli with fruits and oil seed	24.28	0.71		x		n.d.			x	n.d.	n.d.			x	n.d.
milk chocolate	34.02	0.53		x					x	n.d.	n.d.			x	
wafer with vanila cream	25.94	0.09		x		n.d.			x	n.d.	n.d.		x		n.d.
pesto genovese	30.13	0.28		x		n.d.			x	n.d.	n.d.			x	n.d.
pistachio icecream	34.18	0.08		x			x			23.98	23.7		x		
										24.19	23.93				
pistachio cream	23.47	0.20		x			x			20.48	20.23			x	n.d.
										20.16	20.67				
rustic slice	29.57	0.16			x	n.d.	x			20.11	19.7			x	
										20.09	19.74				
roasted and salted pistachio	29.22	0.15		x			x			26.56	26.35			x	n.d.
										27.71	26.54				
pistachio yogurt	n.d.	n.d.		x			x			30.74	30.36			x	
										30.75	30.19				
"tarallucci" snack	23.95	0.66			x	n.d.		x		**37.15**	**37.7**		x		
										**38.02**	**36.76**				
biscuits	29.41	0.14			x	n.d.		x		n.d.	**39**			x	
											**37.15**				
4 nuts cream	35.54	2.62			x			x						x	
rice snack	31.27	1.34	x			34.75		x					x		
						35.05									
breadstick	24.39	0.60	x			21.53		x					x		
						22.22									
snack bar	28.60	3.24	x			20.99			x					x	
						20.27									
tofu	26.78	1.04	x			27.76		x					x		
						28.06									
integral cereals	23.20	1.03	x			24.57			x					x	
						26.00									
tarallucci salt snack (SS)	27.31	0.62													27.25
															28.08
breadstick (SS)	23.64	0.57													24.51
															25.28
integral toasted bread (SS)	24.75	0.59													25.11
															25.81
wafer with vanilla cream (SS)	24.33	0.41													24.78
															25.29
cereal biscuits filled with yogurt (SS)	24.47	1.14													25.38
															26.54

SS: spiked samples with macadamia nut; DiL: declared on label; NDiL: not declared on label; PAL: precautionary allergen labelling; Cq: quantification cycle; SDr: repeatability standard deviation; ΔCq: difference between undiluted DNA target and its dilution 1:4; x: PAL referring to nuts; n.d.: not detected; in bold: unexpected amplification signals; (a) and (b): pistachio systems.

**Table 3:** Exclusivity and inclusivity panel and specificity test results.

Sample	Family Name	Scientific Name	Sesame	Pistachio (a–b)	Macadamia Nut
cashew nut	Anacardiaceae	*Anacardium occidentale*	n.d.	n.d.	n.d.
peanut	Fabaceae	*Arachis hypogaea*	n.d.	n.d.	n.d.
oat	Poaceae	*Avena sativa*	n.d.	n.d.	n.d.
crustacean	Nephropidae	*Nephrops norvegicus*	n.d.	n.d.	n.d.
spelt	Poaceae	*Triticum monococcum*	n.d.	n.d.	n.d.
wheat	Poaceae	*Triticum aestivum*	n.d.	n.d.	n.d.
wheat	Poaceae	*Triticum duro*	n.d.	n.d.	n.d.
kamut	Poaceae	*Triticum turgidum*	n.d.	n.d.	n.d.
cow	Bovidae	*Bos taurus*	n.d.	n.d.	n.d.
lupine	Fabaceae	*Lupinus albus*	n.d.	n.d.	n.d.
almond	Rosaceae	*Prunus dulcis*	n.d.	n.d.	n.d.
molluscs	Octopodidae	*Octopus vulgaris*	n.d.	n.d.	n.d.
hazelnut	Betulaceae	*Corylus avellana*	n.d.	n.d.	n.d.
walnut	Juglandaceae	*Juglans regia*	n.d.	n.d.	n.d.
Brazil nut	Lecythidaceae	*Bertholletia excelsa*	n.d.	n.d.	n.d.
macadamia nut	Proteaceae	*Macadamia intergrifolia*	n.d.	n.d.	22.5 Cq
pecan nut	Juglandaceae	*Carya illinoinensis*	n.d.	n.d.	n.d.
barley	Poaceae	*Hordeum vulgare*	n.d.	n.d.	n.d.
fish	Salmonidae	*Salmo salar*	n.d.	n.d.	n.d.
pine nut	Pinaceae	*Pinus pinea*	n.d.	n.d.	n.d.
pistachio	Anacardiaceae	*Pistacia vera*	n.d.	20.3 Cq	n.d.
rice	Poaceae	*Oryza sativa*	n.d.	n.d.	n.d.
celery	Apiacea	*Apium graveolens*	n.d.	n.d.	n.d.
rye	Poaceae	*Secale cereale*	n.d.	n.d.	n.d.
linseed	Linaceae	*Linum usitatissimum*	n.d.	n.d.	n.d.
mustard	Brassicaceae	*Brassica alba*	n.d.	n.d.	n.d.
sesame	Pedaliaceae	*Sesamum indicum*	21.6 Cq	n.d.	n.d.
soybean	Fabaceae	*Glycine max*	n.d.	n.d.	n.d.
chicken	Phasianidae	*Gallus gallus*	n.d.	n.d.	n.d.
pink pepper	Anacardiaceae	*Schinus molle*	n.t.	n.d.	n.t.
mango	Anacardiaceae	*Mangifera indica*	n.t.	n.d.	n.t.

n.t.: not tested; n.d.: not detected; Cq: quantification cycle; (a–b): pistachio (a) and pistachio (b) systems.

**Table 4:** Limit of detection (LOD) results for sesame, pistachio (a–b) and macadamia nut systems.

Allergen System	LOD_10_						LOD_95_ pos/60
	ng	pos	mean Cq	SDr (Cq)	ΔCq	RSDr % (ng)	
sesame	0.310	10/10	30.70	0.16	1.20	9.63	
	0.155	10/10	31.90	0.21	1.19	12.77	
	0.078	10/10	33.08	0.19	1.18	11.63	
	0.039	10/10	34.26	0.19	0.94	11.69	
	0.019	10/10	35.20	0.20	1.01	11.47	
	0.010	10/10	36.21	0.47	1.48	26.73	
	0.005	10/10	37.68	0.45	0.58	28.26	60/60
	0.002	10/10	38.27	0.58	0.66	33.87	57/60
	0.001	9/10	38.92	0.65	n.v.	n.v.	
pistachio_a	0.120	10/10	30.47	0.13	0.90	8.72	
	0.060	10/10	31.37	0.14	0.92	9.37	
	0.030	10/10	32.29	0.28	1.12	17.86	
	0.015	10/10	33.41	0.52	1.11	34.13	
	0.008	10/10	34.52	0.50	0.82	32.87	
	0.004	10/10	35.33	0.67	1.72	35.23	59/60
	0.002	10/10	37.06	1.07	0.76	72.62	54/60
	0.001	7/10	37.82	1.03	0.15	n.v.	
	0.0005	5/10	37.97	1.24	n.v.	n.v.	
pistachio_b	0.120	10/10	29.92	0.15	1.13	9.10	
	0.060	10/10	31.05	0.15	1.08	8.81	
	0.030	10/10	32.13	0.30	1.07	16.90	
	0.015	10/10	33.20	0.26	1.54	15.86	
	0.008	10/10	34.74	0.77	0.94	36.55	60/60
	0.004	7/10	35.68	0.70	1.28	n.v.	60/60
	0.002	5/10	36.96	0.66	1.30	n.v.	52/60
	0.001	3/10	38.25	0.84	n.v.	n.v.	
	0.0005	0/10	n.v.	n.v	n.v.	n.v.	
macadamia nut	0.800	10/10	29.35	0.07	1.12	4.25	
	0.400	10/10	30.47	0.08	0.96	5.34	
	0.200	10/10	31.43	0.14	1.33	8.67	
	0.100	10/10	32.76	0.23	0.98	15.49	
	0.050	10/10	33.74	0.23	1.07	14.54	
	0.025	10/10	34.81	0.52	1.06	25.95	
	0.013	10/10	35.87	0.49	1.57	30.08	
	0.006	10/10	37.45	0.73	0.39	37.15	60/60
	0.003	8/10	37.83	0.90	n.v.	n.v.	56/60

Cq: quantification cycle; SDr: repeatability standard deviation; RSDr: relative repeatability standard deviation; n.v.: no value; pos: positive amplification signals; LOD_10_: results of 10 replicates for dilution; LOD_95_: results of 60 replicates for the dilution of interest; ΔCq: difference between mean Cq; (a–b): pistachio (a) and pistachio (b) systems.

**Table 5:** Robustness results for sesame, pistachio (a–b), and macadamia nut systems.

Ta	run 1	run 2	
	60 °C	61 °C	
Allergen system		A	B
	Cq	Cq	Cq
sesame	35.1	35.0	35.0
	35.2	34.5	35.0
	34.8	34.9	34.6
pistachio_a	33.7	31.5	31.6
	34.4	31.1	30.5
	33.3	31.5	31.4
pistachio_b	33.5	30.9	30.0
	33.4	32.5	30.3
	33.2	31.2	29.6
macadamia nut	35.1	35.7	36.4
	35.4	35.0	35.3
	35.5	35.2	35.0

Cq: quantification cycle; A: +1 µL reaction volume; B: −1 µL reaction volume; run 1: 7900HT Fast Real-Time PCR System; run 2: 7500 Fast Real-Time PCR System; Ta: annealing temperature; (a–b): pistachio (a) and pistachio (b) systems.

## Supplementary Materials

The following are available online at https://www.mdpi.com/2304-8158/9/8/1085/s1. Supplementary Table S1: Inhibition test for allergen targets and samples used for specificity, Supplementary Table S2: Data of mean Cq, repeatability standard deviation (SDr) and ΔRn for sesame, macadamia nut and pistachio (a–b) of primer and probe optimisation assays, Supplementary Table S3: Data relative to LOD_95_ for sesame, macadamia nut and pistachio a–b.

## References

[1] Manea I., Ailenei E., Deleanu D. (2016). Overview of food allergy diagnosis. Clujul Med..

[2] Tang M.L., Mullins R.J. (2017). Food allergy: Is prevalence increasing?. Intern. Med. J..

[3] Prescott S., Allen K.J. (2011). Food allergy: Riding the second wave of the allergy epidemic. Pediatr. Allergy Immunol..

[4] Sicherer S.H., Sampson H.A. (2014). Food allergy: Epidemiology, pathogenesis, diagnosis, and treatment. J. Allergy Clin. Immunol..

[5] Orruño E., Morgan M.R. (2006). IgE binding to proteins from sesame and assessment of allergenicity: Implications for biotechnology?. Biotechnol. Lett..

[6] Zhang W.J., Cai Q., Guan X., Chen Q. (2015). Detection of peanut (*Arachis hypogaea*) allergen by Real-time PCR method with internal amplification control. Food Chem..

[7] Linacero R., Sanchiz A., Ballesteros I., Cuadrado C. (2019). Application of real-time PCR for tree nut allergen detection in processed foods. Crit. Rev. Food Sci. Nutr..

[8] European Parliament, European Commission (2011). Regulation (EU) No 1169/2011 of the European Parliament and of the Council of 25 October 2011 On the Provision of Food Information to Consumers, Amending Regulations (EC) No 1924/2006 and (EC) No 1925/2006 of the European Parliament and of the Council, and Repealing Commission Directive 87/250/EEC, Council Directive 90/496/EEC, Commission Directive 1999/10/EC, Directive 2000/13/EC of the European Parliament and of the Council, Commission Directives 2002/67/EC and 2008/5/EC and Commission Regulation (EC) No 608/2004.

[9] De la Cruz S., Lopez-Calleja I., Martín R., Gonzalez I., Alcocer M., García T., Lin J., Alcocer M. (2017). Recent advances in the detection of allergens in foods. Food Allergens. Methods in Molecular Biology.

[10] Monaci L., Visconti A. (2010). Immunochemical and DNA-based methods in food allergen analysis and quality assurance perspectives. Trends Food Sci. Technol..

[11] Koeberl M., Clarke D., Lopata A.L. (2014). Next generation of food allergen quantification using mass spectrometric systems. J. Proteome Res..

[12] Török K., Hajas L., Horváth V., Schall E., Bugyi Z., Kemény S., Tömösközi S. (2015). Identification of the factors affecting the analytical results of food allergen ELISA methods. Eur. Food Res. Technol..

[13] Do A.B., Khuda S.E., Sharma G.M. (2018). Undeclared food allergens and gluten in commercial food products analyzed by ELISA. J. AOAC Int..

[14] Chung Y.C., William N., Kerry O., Eric A.E.G. (2015). Multiplex detection of food allergens and gluten. Anal. Bioanal. Chem..

[15] Planque M., Arnould T., Renard P., Delahaut P., Dieu M., Gillard N. (2017). Highlight on bottlenecks in food allergen Analysis: Detection and quantification by mass spectrometry. J. AOAC Int..

[16] Salihah N.T., Hossain M.M., Lubis H., Ahmed M.U. (2016). Trends and advances in food analysis by real-time polymerase chain reaction. J. Food Sci. Technol..

[17] Holzhauser T., Oliver S., Vieths S., Koppelman S.J., Hefle S.L. (2006). Polymerase chain reaction (PCR) methods for the detection of allergenic foods. Detecting Allergens in Food.

[18] Pierboni E., Rondini C., Torricelli M., Ciccone L., Tovo G.T., Mercuri M.L., Altissimi S., Haouet N. (2018). Digital PCR for analysis of peanut and soybean allergens in foods. Food Control.

[19] Redl G., Husain F.T., Bretbacher I.E., Nemes A., Cichna-Markl M. (2010). Development and validation of a sandwich ELISA for the determination of potentially allergenic sesame (*Sesamum indicum*) in food. Anal. Bioanal. Chem..

[20] López-Calleja I.M., de la Cruz S., Martín R., González I., García T. (2015). Duplex real-time PCR method for the detection of sesame (*Sesamum indicum*) and flaxseed (*Linum usitatissimum*) DNA in processed food products. Food Addit. Contam. Part A Chem. Anal. Control Expo. Risk Assess..

[21] EFSA (2014). Scientific opinion on the evaluation of allergenic foods and food ingredients for labeling purposes. EFSA J..

[22] Kägi M.K., Wüthrich B. (1993). Falafel burger anaphylaxis due to sesame seed allergy. Ann. Allergy.

[23] Kanny G., De Hauteclocque C., Moneret-Vautrin D.A. (1996). Sesame seed and sesame seed oil contain masked allergens of growing importance. Allergy.

[24] Adatia A., Clarke A.E., Yanishevsky Y., Ben-Shoshan M. (2017). Sesame allergy: Current perspectives. J. Asthma Allergy.

[25] Vocks E., Borga A., Szliska C., Seifert H.U., Seifert B., Burow G., Borelli S. (1993). Common allergenic structures in hazelnut, rye grain, sesame seeds, kiwi, and poppy seeds. Allergy.

[26] Schöringhumer K., Redl G., Cichna-Markl M. (2009). Development and validation of a duplex real-time PCR method to simultaneously detect potentially allergenic sesame and hazelnut in food. J. Agric. Food Chem..

[27] Pastorello E.A., Varin E., Farioli L., Pravettoni V., Ortolani C., Trambaioli C., Fortunato D., Gabriella Giuffrida M., Rivolta F., Robino A. (2001). The major allergen of sesame seeds (*Sesamum indicum*) is a 2S albumin. J. Chromatogr..

[28] Beyer K., Bardina L., Grishina G., Sampson H.A. (2002). Identification of sesame seed allergens by 2-dimensional proteomics and Edman sequencing: Seed storage proteins as common food allergens. J. Allergy Clin. Immunol..

[29] Leduc V., Moneret-Vautrin D.A., Tzen J.T.C., Morisset M., Guerin L., Kanny G. (2006). Identification of oleosins as major allergens in sesame seed allergic patients. Allergy.

[30] Beyer K., Grishina G., Bardina L., Sampson H.A. (2007). Identification of 2 new sesame seed allergens: Ses i 6 and Ses i 7. J. Allergy Clin. Immunol..

[31] Brezinski L.J. (2007). Detection of sesame seed DNA in foods using real-time PCR. J. Food. Prot..

[32] Schöringhumer K., Cichna-Markl M. (2007). Development of a real-time PCR method to detect potentially allergenic sesame (*Sesamum indicum*) in food. J. Agric. Food Chem..

[33] Mustorp S., Engdahl-Axelsson C., Svensson U., Holck A. (2008). Detection of celery (*Apium graveolens*), mustard (*Sinapis alba*, *Brassica juncea*, *Brassica nigra*) and sesame (*Sesamum indicum*) in food by real-time PCR. Eur. Food Res. Technol..

[34] Köppel R., Dvorak V., Zimmerli F., Breitenmoser A., Eugster A., Waiblinger H.U. (2010). Two tetraplex real-time PCR for the detection and quantification of DNA from eight allergens in food. Eur. Food Res. Technol..

[35] Köppel R., van Velsen-Zimmerli F., Bucher T. (2012). Two quantitative hexaplex real-time PCR systems for the detection and quantification of DNA from twelve allergens in food. Eur. Food Res. Technol..

[36] Zhang W., Zhao Y., Xu Qingjin C.Q. (2018). Development of a triplex real-time PCR for simultaneous detection of allergenic ingredients in processed food. Food chemistry and safety. Czech J. Food Sci..

[37] Ehlert A., Demmel A., Hupfer C., Busch U., Engel K.H. (2009). Simultaneous detection of DNA from 10 food allergens by ligation-dependent probe amplification. Food Addit. Contam. Part A Chem. Anal. Control Expo. Risk Assess..

[38] López-Calleja I.M., García A., Madrid R., García T., Rosario M., González I. (2017). Multiplex ligation-dependent probe amplification (MLPA) for simultaneous detection of DNA from sunflower, poppy, flaxseed, sesame and soy allergenic ingredients in commercial food products. Food Control.

[39] Sanchiz A., Ballesteros I., Martin A., Rueda J., Pedrosa M.M., Dieguez M.d.C., Rovira M., Cuadrado C., Linacero R. (2017). Detection of pistachio allergen coding sequences in food products: A comparison of two real time PCR approaches. Food Control.

[40] Noorbakhsh R., Mortazavi S.A., Sankian M., Shahidi F., Assarehzadegan M.A., Varasteh A. (2010). Cloning, expression, characterization, and computational approach for cross-reactivity prediction of manganese superoxide dismutase allergen from pistachio nut. Allergol. Int..

[41] Roux K.H., Teuber S.S., Sathe S.K. (2003). Tree nut allergens. Int. Arch. Allergy Immunol..

[42] Costa J., Silva I., Vicente A.A., Oliveira M.B.P.P., Mafra I. (2019). Pistachio nut allergy: An updated overview. Crit. Rev. Food Sci. Nutr..

[43] Barbieri G., Frigeri G. (2006). Identification of hidden allergens: Detection of pistachio traces in mortadella. Food Addit. Contam..

[44] López-Calleja I.M., de la Cruz S., González I., Rosario M.T.G. (2013). Survey of undeclared allergenic pistachio *(Pistacia vera)* in commercial foods by hydrolysis probe real-time PCR. Food Control.

[45] Cheng F., Wu J., Zhang J., Pan A., Quan S., Zhang D., Kim H., Li X., Zhou S., Yang L. (2016). Development and inter-laboratory transfer of a decaplex polymerase chain reaction assay combined with capillary electrophoresis for the simultaneous detection of ten food allergens. Food Chem..

[46] Mast A.R., Willis C.L., Jones E.H., Downs K.M., Weston P.H. (2008). A smaller Macadamia from a more vagile tribe: Inference of phylogenetic relationships, divergence times, and diaspore evolution in Macadamia and relatives (tribe Macadamieae; Proteaceae). Am. J. Bot..

[47] Brežná B., Piknová L., Kuchta T. (2009). A novel real-time polymerase chain reaction method for the detection of macadamia nuts in food. Eur. Food Res. Technol..

[48] Ito M., Mizota T., Kitaguchi T., Ohno K., Ohba T., Tanaka M. (2018). Simultaneous detection of eight species of tree nut in foods using two tetraplex polymerase chain reaction assays. Biosci. Biotechnol. Biochem..

[49] European Commission (2014). EUR 27021 EN. Guidelines for Sample Preparation Procedures in GMO Analysis.

[50] ISO (2013). ISO 21571:2013. Foodstuffs—Methods of Analysis for the Detection of Genetically Modified Organisms and Derived Products—Nucleic Acid Extraction.

[51] de Ronde M.W.J., Ruijter J.M., Lanfear D., Bayes-Genis A., Kok M.G.M., Creemers E.E., Pinto Y.M., Pinto-Sietsma S.J. (2017). Practical data handling pipeline improves performance of qPCR-based circulating miRNA measurements. RNA.

[52] Waiblinger H.U., Grohmann L. (2014). Guidelines for validation of DNA extraction methods applied in subsequent PCR analysis of food and feed products for the presence of genetically modified material. J. Verbrauch. Lebensm..

[53] European Commission (2017). EUR 29015 EN. Verification of Analytical Methods for GMO Testing when Implementing Interlaboratory Validated Methods.

[54] Torricelli M., Pierboni E., Tovo G.R., Rondini C. (2016). In-House validation of a DNA extraction protocol from honey and bee pollen and analysis in fast real-time PCR of commercial honey samples using a knowledge-based approach. Food Anal. Methods.

[55] C-Value Introduction to the Plant DNA C-Vaues Database. https://cvalues.science.kew.org.

[56] Arumuganathan K., Earle E.D. (1991). Nuclear DNA content of some important plant species. Plant Mol. Biol. Report..

[57] Broeders S., Huber I., Grohmann L., Berben G., Taverniers I., Mazzara M., Roosens N., Morisset D. (2014). Guidelines for validation of qualitative real-time PCR methods. Trends Food Sci. Technol..

[58] Pierboni E., Curcio L., Tovo G.R., Torricelli M., Rondini C. (2016). Evaluation of systems for nopaline synthase terminator in fast and standard real-time PCR to screen genetically modified organisms. Food Anal. Methods.

[59] European Commission (2015). JRC95544. Definition of Minimum Performance Requirements for Analytical Methods of GMO Testing.

[60] Huggett J.F., Foy C.A., Benes V., Emslie K., Garson J.A., Haynes R., Hellemans J., Kubista M., Mueller R.D., Nolan T. (2013). The digital MIQE guidelines: Minimum information for publication of quantitative digital PCR experiments. Clin. Chem..

[61] Köppel R., Bucher T. (2015). Rapid establishment of droplet digital PCR for quantitative GMO analysis. Eur. Food Res. Technol..

