# Supplementary material for: Sesame, Pistachio, and Macadamia Nut: Development and Validation of New Allergenic Systems for Fast Real-Time PCR Application
Source: Foods. 2020 Aug 8;9(8):1085. doi: 10.3390/foods9081085 (PMC7464690; doi:10.3390/foods9081085)
Supplement: Supplementary file 1 [file foods-09-01085-s001.pdf]

Supplementary Table S1. Inhibition test for allergen targets and samples used for specificity.

sample	family name	scientific name	actin system	
			**Cq**	**ΔCq**
sesame*	Pedaliaceae	*Sesamum indicum*	26.50	1.79
pistachio*	Anacardiaceae	*Pistacia vera*	28.26	2.12
macadamia nut*	Proteaceae	*Macadamia intergrifolia*	22.64	2.29
cashew nut	Anacardiaceae	*Anacardium occidentale*	27.85	1.53
peanut	Fabaceae	*Arachis hypogaea*	28.94	2.12
oat	Poaceae	*Avena sativa*	23.64	2.16
spelt	Poaceae	*Triticum monococcum*	25.55	2.35
wheat	Poaceae	*Triticum aestivum*	23.32	2.10
wheat	Poaceae	*Triticum duro*	23.48	2.11
kamut	Poaceae	*Triticum turgidum*	23.37	2.06
lupine	Fabaceae	*Lupinus albus*	23.40	2.09
almond	Rosaceae	*Prunus dulcis*	32.60	2.24
hazelnut	Betulaceae	*Corylus avellana*	31.15	2.01
walnut	Juglandaceae	*Juglans regia*	28.47	1.79
Brazil nut	Lecythidaceae	*Bertholletia excelsa*	24.83	1.89
Pecan nut	Juglandaceae	*Carya illinoinensis*	27.93	2.10
barley	Poaceae	*Hordeum vulgare*	23.88	2.13
pine nut	Pinaceae	*Pinus pinea*	28.94	1.92
rice	Poaceae	*Oryza sativa*	27.15	2.12
celery	Apiacea	*Apium graveolens*	25.96	2.25
rye	Poaceae	*Secale cereale*	26.63	2.00
linseed	Linaceae	*Linum usitatissimum*	24.74	1.74
mustard	Brassicaceae	*Brassica alba*	24.20	1.96
soybean	Fabaceae	*Glycine max*	21.99	2.11
pink pepper	Anacardiaceae	*Schinus molle*	29.10	1.77
mango	Anacardiaceae	*Mangifera indica*	25.60	2.25

Cq: quantification cycle; ΔCq: difference between undiluted DNA target and its dilution 1:4; *: allergen targets of this study.

Supplementary Table S2. Data of mean Cq, repeatability standard deviation (SDr) and ΔRn for sesame, macadamia nut and pistachio (a-b) of primer and probe optimization assays.

sesame			
	mean Cq	SDr	ΔRn
150F/150R	33.19	0.17	3000
300F/150R	32.61	0.15	3000
900F/150R	31.99	0.10	3500
150F/300R	32.98	0.09	3500
300F/300R	32.09	0.13	4000
900F/300R	31.61	0.10	4500
150F/900R	32.71	0.08	3800
300F/900R	31.90	0.11	4500
**900F/900R**	**31.33**	**0.16**	**5800**
100P	32.02	0.07	3000
**150P**	**31.66**	**0.13**	**3800**
200P	31.62	0.14	4500
250P	31.59	0.08	5800

macadamia nut			
	mean Cq	SDr	ΔRn
150F/150R	32.76	0.14	1800
300F/150R	32.80	0.16	1800
900F/150R	33.30	0.19	1800
150F/300R	32.59	0.20	1800
300F/300R	32.53	0.20	2500
900F/300R	33.07	0.16	2500
150F/900R	32.43	0.21	2500
300F/900R	32.55	0.25	2500
**900F/900R**	**32.79**	**0.23**	**2500**
100P	33.74	0.25	1500
150P	33.59	0.11	1500
200P	33.02	0.23	2500
**250P**	**32.84**	**0.10**	**2500**

pistachio_a			
	mean Cq	SDr	ΔRn
150F/150R	27.30	0.03	4500
300F/150R	27.08	0.03	5400
**900F/150R**	**27.04**	**0.05**	**5800**
150F/300R	27.52	0.03	3800
300F/300R	27.32	0.01	4700
900F/300R	27.21	0.02	5400
150F/900R	27.88	0.02	3600
300F/900R	27.66	0.03	4400
900F/900R	27.58	0.03	5200
100P	27.68	0.03	2500
150P	27.25	0.04	3700
**200P**	**27.08**	**0.05**	**4600**
250P	26.92	0.05	5800

pistachio_b			
	mean Cq	SDr	ΔRn
150F/150R	26.87	0.05	3800
300F/150R	26.48	0.07	5200
**900F/150R**	**26.32**	**0.02**	**6000**
150F/300R	27.18	0.05	3400
300F/300R	26.78	0.05	4500
900F/300R	26.63	0.05	5100
150F/900R	27.61	0.08	3500
300F/900R	27.29	0.01	4100
900F/900R	27.04	0.04	5000
100P	26.80	0.01	2200
150P	26.48	0.02	3300
**200P**	**26.37**	**0.03**	**4600**
250P	26.26	0.03	6000

*in bold: concentration (nM) of primers and probe chosen for the allergen targets (sesame, macadamia nut, pistachio a-b), corresponding to the best values in terms of highest ΔRn, minor Cq and lowest standard deviation; F; primer forward; R: primer reverse; P: probe; Cq: quantification cycle; SDr: repeatability standard deviation; ΔRn: normalized reporter fluorescence signal.

Supplementary Table S3. Data relative to LOD_{95} for sesame, macadamia nut and pistachio a-b.

**LOD_{95} sesame**							**LOD_{95} pistachio_a**						
**ng**	**Cq**						**ng**	**Cq**					
	37.92	36.63	39.56	36.58	36.85	37.79		35.98	36.87	37.19	35.41	36.41	36.98
	36.78	38.31	36.76	36.56	38.31	37.68		34.73	35.98	36.04	36.17	34.94	35.35
	37.57	37.62	38.01	36.45	37.50	36.64		35.02	37.00	35.33	35.03	34.59	n.d.
	37.64	37.01	37.74	36.69	36.66	38.25		37.00	34.56	37.39	35.99	37.25	36.96
0.002	36.45	39.24	37.51	37.82	39.69	36.97	0.002	n.d.	35.47	n.d.	35.99	36.01	36.89
	36.77	n.d.	36.85	37.93	37.32	37.29		34.98	36.14	36.88	36.54	37.63	n.d.
	n.d.	36.77	38.62	n.d.	36.80	37.25		35.90	36.12	35.44	35.97	36.94	36.07
	36.29	36.27	37.18	37.94	36.73	38.28		37.22	n.d.	35.00	n.d.	36.53	35.99
	37.72	36.54	36.67	39.49	37.71	37.55		37.08	35.37	35.69	35.43	37.34	35.53
	37.65	38.49	39.48	37.81	37.62	38.49		35.95	35.44	35.85	35.46	35.59	35.98
	35.59	35.38	36.20	37.37	36.48	37.29		35.05	34.81	35.89	35.03	34.55	34.44
	36.69	36.26	35.70	36.53	36.40	36.73		34.41	33.96	34.80	34.55	35.46	35.50
	35.81	36.13	35.86	36.85	36.20	36.60		34.15	35.49	34.18	35.22	34.98	34.54
	36.65	36.08	36.12	36.21	35.86	36.36		34.64	34.84	34.42	34.49	35.08	35.11
0.005	35.93	36.60	36.18	36.65	36.39	36.90	0.004	34.26	35.34	36.46	34.79	35.22	34.90
	35.38	35.96	36.90	36.41	37.82	36.50		34.86	34.07	36.09	34.69	34.86	35.13
	35.97	35.73	36.63	38.51	36.77	37.00		36.04	n.d.	35.20	34.50	34.85	35.17
	36.25	35.27	38.15	36.79	35.77	37.98		34.75	35.13	35.80	34.70	34.31	34.90
	35.75	36.17	36.55	36.95	36.40	36.45		34.89	34.47	35.02	34.78	35.41	35.13
	36.01	36.30	36.35	37.90	36.31	36.58		34.06	36.05	34.57	34.34	34.17	35.17
**LOD_{95} macadamia nut**							**LOD_{95} pistachio_b**						
**ng**	**Cq**						**ng**	**Cq**					
	36.27	35.44	35.43	36.46	36.50	36.28		34.77	36.27	35.44	33.57	35.35	35.47
	35.64	37.29	35.69	36.93	35.64	36.11		36.18	34.74	34.70	36.57	35.17	33.64
	35.82	37.13	37.17	35.70	35.82	36.04		34.58	35.42	36.66	34.18	35.38	34.93
	36.60	35.63	36.90	35.86	36.68	36.58		34.35	34.04	35.62	35.45	36.39	35.45
0.006	36.67	35.82	37.88	35.86	35.21	35.89	0.004	35.35	34.22	33.46	36.19	34.87	33.68
	36.58	37.56	36.45	35.95	35.90	36.35		34.81	36.09	35.26	36.63	34.19	35.31
	36.94	37.08	36.82	35.23	36.48	36.85		35.31	34.27	34.96	35.16	34.77	35.31
	35.82	37.06	37.23	36.59	36.55	36.48		34.47	34.25	34.21	36.67	35.29	36.51
	39.34	36.09	35.84	35.67	36.58	36.00		34.49	34.47	33.54	34.15	34.82	34.63
	37.22	36.80	36.56	35.51	35.79	37.10		34.72	34.86	33.43	34.19	37.06	35.14
	37.45	36.91	37.11	38.34	36.91	37.35		35.02	35.57	n.d.	36.00	34.85	35.92
	37.24	36.21	n.d.	36.31	38.53	36.17		36.90	35.98	37.42	35.98	n.d.	37.70
	36.04	37.06	37.08	36.59	37.70	36.80		n.d.	34.71	n.d.	36.20	35.31	36.11
	35.78	n.d.	36.79	36.87	36.95	37.08		34.91	37.83	35.98	38.24	36.50	37.12
0.003	36.74	36.18	n.d.	37.28	37.34	36.86	0.002	35.25	n.d.	36.36	35.90	36.47	35.98
	36.00	35.39	35.85	37.19	38.13	37.42		35.57	37.54	36.25	34.82	37.51	35.71
	36.47	37.80	37.72	37.11	37.03	35.47		37.67	35.97	n.d.	35.11	35.35	36.46
	39.03	36.03	36.62	38.43	n.d.	36.53		34.95	35.96	36.07	35.50	35.57	34.69
	37.27	35.79	37.94	36.45	37.38	35.73		35.56	34.75	n.d.	35.43	36.72	35.52
	37.68	37.30	37.52	35.67	35.67	36.62		35.59	n.d.	37.41	36.13	37.46	34.95

LOD_{95}: results of 60 replicates for the dilution of interest; Cq: quantification cycle; n.d.: not detected.
